# Probiotics, Pre-biotics and Synbiotics in the Treatment of Pre-diabetes: A Systematic Review of Randomized Controlled Trials

**DOI:** 10.3389/fpubh.2021.645035

**Published:** 2021-03-26

**Authors:** Xian Wang, Jiao Yang, Xianliang Qiu, Qing Wen, Min Liu, Dongqi Zhou, Qiu Chen

**Affiliations:** ^1^School of Clinical Medicine, Chengdu University of Traditional Chinese Medicine, Chengdu, China; ^2^Department of Endocrinology, Hospital of Chengdu University of Traditional Chinese Medicine, Chengdu, China

**Keywords:** pre-diabetes, probiotics, pre-biotics, synbiotics, gut microbiota

## Abstract

**Objectives:** This study aimed to review the data from randomized controlled trials (RCTs) and identify evidence for microbiota's role and use of probiotics, pre-biotics, or synbiotics in pre-diabetes.

**Methods:** RCTs of pro-, pre-, synbiotics for the treatment of pre-diabetes population will be summarized. We searched for EMBASE, MEDLINE, Web of Science, Cochrane Central, Clinical Trials (ClinicalTrials.gov) from inception to February 2021.

**Results:** The gut microbiota influences host metabolic disorders via the modulation of metabolites, including short-chain fatty acids (SCFAs), the endotoxin lipopolysaccharides (LPS), bile acids (BA) and trimethylamine N-oxide (TMAO), as well as mediating the interaction between the gastrointestinal system and other organs. Due to the limited sources of studies, inconsistent outcomes between included studies. Probiotics can decrease glycated hemoglobin (HbA1c) and have the potential to improve post-load glucose levels. The supplementation of probiotics can suppress the rise of blood cholesterol, but the improvement cannot be verified. Pre-biotics are failed to show an evident improvement in glycemic control, but their use caused the changes in the composition of gut microbiota. A combination of probiotics and pre-biotics in the synbiotics supplementation is more effective than probiotics alone in glycemic control.

**Conclusion:** In the current studies using probiotics, pre-biotics or synbiotics for the treatment of pre-diabetes, the benefits of modulating the abundance of gut microbiota were partially demonstrated. However, there is insufficient evidence to show significant benefits on glucose metabolism, lipid metabolism and body composition.

## Introduction

### Diabetes and Pre-diabetes

Diabetes has become a severe health problem worldwide, which occurs along with the raised level of blood glucose. The prevalence of diabetes remains increased, with an estimated 463.0 million adults aged 20–79 years, and the number is expected to reach 578.4 million in 2030 (1). Type 2 diabetes (T2D) is the primary type of diabetes, accounting for ~90% of all cases. The development of T2D can cause different complications, including cardiovascular, eyes, kidney, nerve, and vascular diseases. T2D and these related complications can affect people's life quality and increase expenses on treatment. As a result, the prevention of diabetes is essential by screening, lifestyle intervention and nutrition supplementation, especially for people at high risk.

Pre-diabetes is defined as impaired fasting glucose (IFG), impaired glucose tolerance (IGT), and/or elevated HbA1c levels (2), which are intermediate states between normal glucose homeostasis and diabetes. According to diagnostic criteria from the World Health Organization (WHO), pre-diabetes is defined as IFG: fasting plasma glucose between 110 and 125 mg/dL (5.5–6.9 mmol/L) and/or IGT: 2-h post-load plasma glucose between 140 and 200 mg/dL (7.8–11.0 mmol/L) during a 75 g oral glucose test (3). However, despite the same IGT level for diagnosis, the American Diabetes Association (ADA) applies a lower cut-off value for IFG: FPG between 100 and125 mg/dL (5.5–6.9 mmol/L) and involve glycated hemoglobin (HbA_1_c) between 5.7 and 6.4% as a new diagnostic criterion for pre-diabetes (2). The pathophysiology of pre-diabetes is complex, which is associated with increased glucose levels, decreased insulin sensitivity, increased inflammatory cytokines, and altered incretin responses (4). Insulin resistance at liver and peripheral tissues and defective glucose sensing at the β-cell are the central determinants that together cause and predict hyperglycemia (5). Similar to T2D, people with pre-diabetes may suffer micro-, macrovascular, and neuropathy complications (6).

Due to the uncertain diagnostic criteria for IFG, it is difficult to estimate the trend of pre-diabetes. However, the Centers for Diseases Control and Prevention in the US reported around 84.1 million American adults, or 1 in 3, suffered from pre-diabetes in 2017 (7). Meanwhile, the prevalence of pre-diabetes can be inferred by IGT because it is a unified item in all diagnostic standards. In 2019, the International Diabetes Federation (IDF) reported that 7.5% of the adult population, 373.9 million adults aged 20–79 years, are estimated to have IGT. The number of those people is expected to 453.8 million by 2030 and 548.4 million by 2045 (1). Although the conversion rate is different between nations, ~9.3–55% of people with pre-diabetes converted to T2D within 3 years annually (8). For this reason, it is imperative to prevent or retard the reversible process to T2D among people with pre-diabetes to reduce the burden of T2D.

Previous studies suggest that gut microbiota plays an important role in the development of insulin resistance and diabetes mellitus (9). Gut microbiota can affect metabolic disorders through different potential mechanisms, including modulation of inflammation, gut permeability, glucose metabolism, energy expenditure and fatty acid oxidation and synthesis (10). Therefore, diet-related interventions can be recommended as a useful strategy to control pre-diabetes and prevent or delay T2D by causing beneficial changes in gut microbiota. It has been suggested that oral administration of probiotics, pre-biotics and synbiotics can be an effective method to change gut microbiota composition in pre-diabetes population (11).

### Relationship Between Gut Microbiota and Pre-diabetes

**Figure 1:**
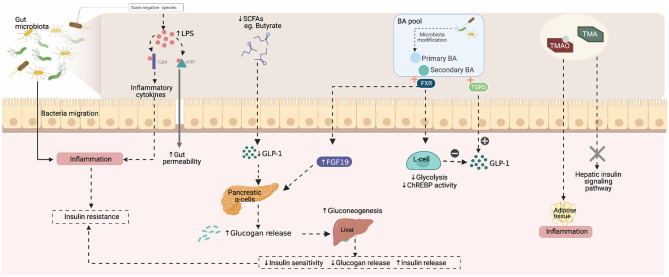
The possible relationship between gut microbiota and pre-diabetes. (Created with BioRender.com).

Gut microbiota is not only digestive but also related to the pathogenesis of many metabolic diseases, such as obesity (12), diabetes (13), non-alcoholic fatty liver (14). Due to the use of genetic factors, lifestyles, antibiotics and changes in diet structure, the diversity of gut microbiota has changed and is characterized by inter-individual variability (15). It plays an important role in T2D and pre-diabetes, including inflammatory response, dietary nutrition, gut permeability, glucose and lipid metabolism, insulin sensitivity and energy homeostasis (10). The possible relationship between gut microbiota and pre-diabetes is shown in Figure 1.

It is reported that the number of bacteria related to short-chain fatty acids (SCFAs) was lower in T2D patients (13). Disturbance of gut microbiota may lead to decreased production of SCFAs, leading to increased inflammatory factors, affecting insulin secretion and sensitivity of islet β cells, and producing insulin resistance (16). SCFAs, especially butyrate, promote the secretion of glucagon-like peptide (GLP)-1, which prevents the secretion of glucagon, inhibits gluconeogenesis in the liver, and improves insulin sensitivity (17). In addition, SCFAs can impede the low-grade inflammation caused by bacteria migration from gut to mesenteric adipose tissue and blood (18). These suggest that the increase of SCFAs, particularly butyrate, are important for preventing and controlling pre-diabetes.

A high-fat diet can increase the percentage of Gram-negative species in gut microbiota (19). Lipopolysaccharides (LPS), a main outer cell membrane component of Gram-negative bacteria, can exist in high concentrations and be absorbed by intestines (20). Furthermore, LPS stimulates the inactive immune system by binding with toll-like receptor (TLR), activating immune cells to release inflammatory cytokines, which promotes insulin resistance caused by an endotoxin-induced inflammatory response (21). Another potential mechanism associated with gut ecosystem homeostasis is the endocannabinoid system. LPS interacts with endocannabinoid receptors (eCB1), modulating gut permeability and LPS translocation, increasing levels of circulating level of LPS and inducing metabolic endotoxemia (22).

Cholic acid and chenodenxycholic acid are primary bile acids (BA) produced by cholesterol in the liver, and primary BA are converted into secondary BA in the intestine (23). Gut microbiota participates in the biotransformation of BA through deconjugation, dehydroxylation, and re-conjugation of BA (24). Moreover, BA is involved in regulating glucose homeostasis as a signaling molecule and cell receptor, directly activating the nuclear farnesoid X receptor (FXR) and the Takeda G protein-coupled receptor 5 (TGR5) signals and indirectly promoting FXR-dependent induction of intestinal fibroblast growth factor-19 (FGF19) (25). TGR5 activation can induce pre-proglucagon gene expression and GLP-1 secretion (17, 26). On the contrary, FXR activation suppresses the pre-proglucagon gene expression and GLP-1 secretion by inhibiting glycolysis and ChREBP activity in L-cells (27). Therefore, in intestinal endocrine L-cells, BA act through the opposite effects on TGR5 and FXR to regulate the production and secretion of GLP-1, thereby maintaining weight loss and improving glucose tolerance.

Gut bacteria metabolize dietary nutrients to produce trimethylamine (TMA), which is then converted to trimethylamine N-oxide (TMAO) in the liver. The previous study has shown that TMAO levels are elevated in T2D patients (28). Also, animal models have shown that dietary TMAO can exacerbate impaired glucose tolerance and increase fasting insulin levels by blocking the hepatic insulin signaling pathway and causing inflammation in adipose tissue (29). Although a prospective study showed that a higher intake of phosphatidylcholine (the pre-cursor for TMAO generation) was independently associated with an increased risk of T2D (30), the association between TMAO and T2D has not reached a consistent conclusion. Roy et al. (31) have observed that plasma TMAO levels are associated with increased prevalence of pre-diabetes in a non-linear fashion but not related to insulin resistance or longitudinal fasting plasma glucose (FPG). The relationship between plasma TMAO and diabetes has not been elucidated, and more researches are needed to explore the development mechanism in the future.

Overall, the gut microbiota influences host metabolic disorders via the modulation of metabolites, including SCFAs, the endotoxin LPS, BA, and TMAO, as well as mediating the interaction between the gastrointestinal system and other organs.

## Study Identification

**Figure 2:**
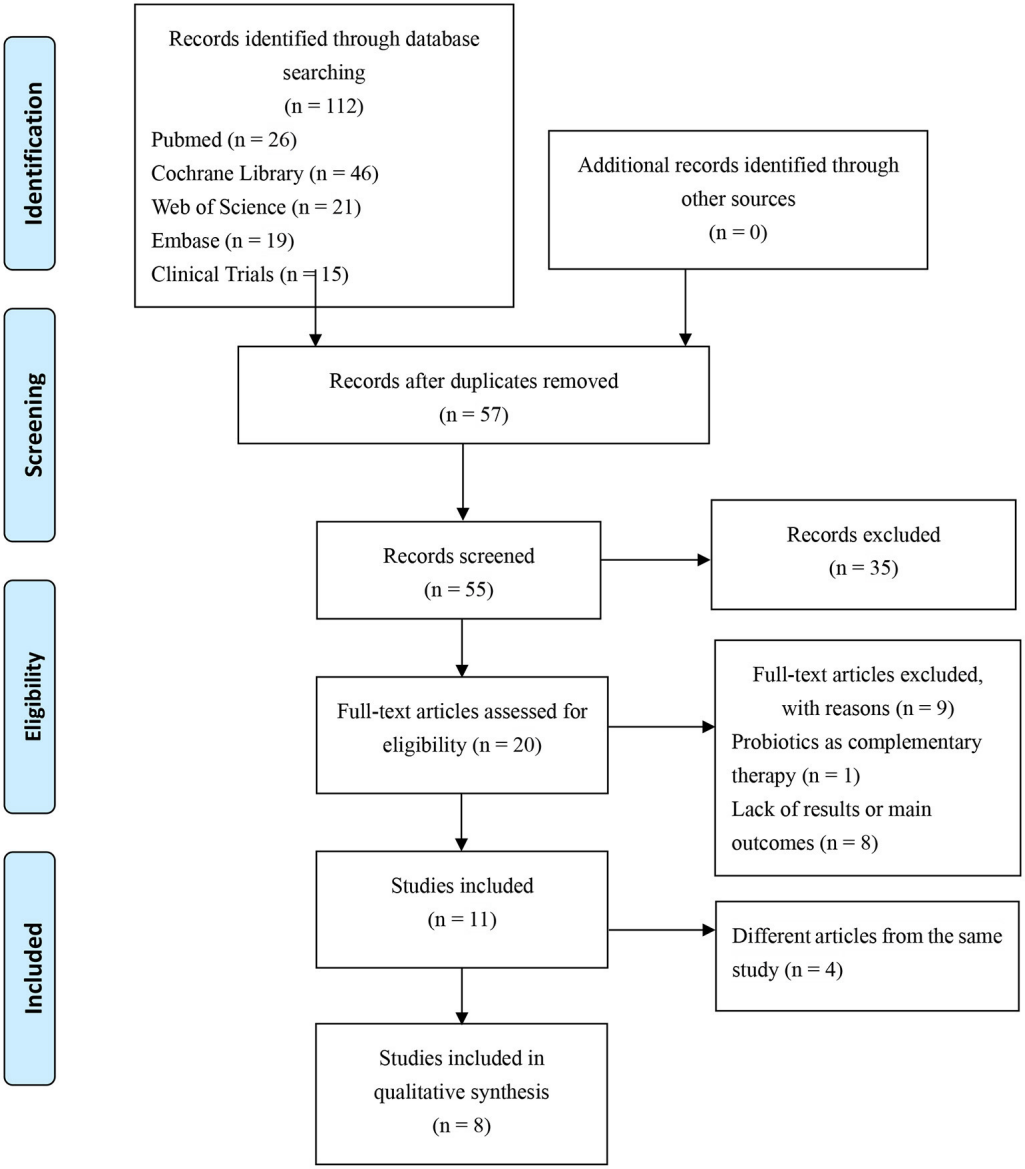
Flow diagram of study selection.

In this review, randomized controlled studies (RCTs) of pro-, pre-, synbiotics for the treatment of pre-diabetes population will be summarized. The following electronic bibliographic databases will be searched from inception to February 2021: EMBASE, MEDLINE, Web of Science, Cochrane Central. Meanwhile, Clinical Trials (ClinicalTrials.gov) will also be searched. A search strategy will be developed using a combination of medical subheadings words and keywords include: “Pre-diabetic State” or “Pre-diabetic States” or “state, Pre-diabetic” or “States, Pre-diabetic” or “Pre-diabetes” and “Probiotics” or “Probiotic” or “Synbiotics” or “Synbiotic” or “Pre-biotics” or “Pre-biotic.” This review will be conducted according to the preferred reporting items for systematic review and meta-analysis protocols (PRISMA-P) 2015 statement (32). The stepwise procedure of the selected studies was shown in the flow diagram of Figure 2. Finally, a total of 8 RCTs are included in the current review, shown in Table 1.

**Table 1:** Human clinical trials of probiotics, pre-biotics, and synbiotics for the treatment of pre-diabetes.

**Study**	**Country**	**No. of patients**	**Blind**	**Inclusion criteria**	**Intervention**	**Control**	**Duration**	**Outcomes**		
								**Anthropometry and body composition**	**Blood biochemical analysis and pancreatic function assessment**	**Fecal compositions and microbiome populations**
Mahboobi et al. (33)	Iran	55	Double	FPG of 100–125 mg/dL, 2hr-glucose tolerance test of 140–200 mg/dL, or both, <2 months	Probiotics: 500 mg/day (7 × 10^9^ CFU *lactobacillus Casei*, 2 × 10^9^ CFU *Lactobacillus Acidophilus*, 1.5 × 10^9^ CFU *Lactobacillus Rhamnosus*, 2 × 10^9^ CFU *Bifidobacterium Breve*, 7 × 10^9^ CFU *Bifidobacterium Longum*, 1.5 × 10^9^ CFU *Streptococcus Thermophilus*)	Placebo: capsules containing starch.	8 weeks	SBP, DBP	TC, TG, HDL-C, LDL-C	N.A
Yang et al. (34)	USA	13	Double	FPG of 100–125 mg/dL and/or HgbA1c of 5.7–6.4%	Pre-biotics: 2 g/day XOS	Placebo	8 weeks	BW, BMI, body fat (%), trunk fat (%)	Glucose^*^, insulin^*^, TG, HOMA-IR^*^, active GLP-1^*^, pancreatic polypeptides^*^, leptin^*^, TNF-α^*^	*Howardella*↓, *Enterorhabdus*↓, *Slackia*↓, *Blautia hydrogenotrophica*↑
Naito et al. (35)	Japan	98	Double	Aged 20 to 64 years; BMI ≥ 25 kg/m^2^; and 1-hr post-load plasma glucose level ≥180 mg/dL	Probiotic: ≥1.0 × 10^11^ CFU LcS (contained in 100 ml milk/day)	Placebo	8 weeks	SBP, DBP, BW, BMI, fat (%)	HbA_1_c, Glucose^‡^, FIL, glycoalbumin, TC↓, TG, HDL-C, LDL-C↓, Non-HDL-C↓, HOMA-IR, HOMA-β, Mastsuda Index, Insulinogenic Index	N.A
Canfora et al. (36)	The Netherlands	44	Double	Aged 45–70 years; BMI of 28–40 kg/m^2^; FPG ≥5.6 mmol/L and/or 2-hr post-load plasma glucose of 7.8–11 mmol/L	Prebiotic: 15 g/d GOS	Placebo: 16.95 g/d maltodextrin	12 weeks	BMI, BW, body fat (% and kg), lean mass (kg), visceral fat (g)	FPG, M-value, HOMA-IR, SCFAs, plasma TAG, plasma free glycerol, plasma FFA, GLP-1, PYY, FIL, leptin, LBP, IL-6, IL-8, TNF-α, energy expenditure, fat oxidation, carbohydrate oxidation	*Bifidobacterium*↑, *Bacteroides stercoris er rel*.↓,*Prevotella oralis et rel*.↑,*Prevotella melaninogenica et rel*.↑,*Sutterella wadsworthia et rel*.↓
Baugh (68)	USA	18	Double	Aged 40–75 years; BMI of 25–40 kg/ m^2^; ADA risk assessment score ≥5, HbA_1_c of 5.7–6.4%, with FPG of 100–125 mg/dL, or 2-hr OGTT value of 140–200 mg/dL	Prebiotic: 10 g/day (inulin, 5 g/day for first 7 days, then move to 10 g/day)	Placebo	6 weeks	N.A	Fasting and postprandial plasma TMAO, TMA moiety concentrations, choline, betaine, L-carnitine, γ-butyrobetaine concentrations	N.A
Kassaian^§^	Iran	120	Double	Aged 35–75 years old; FPG of 100–125 mg/dL or 2-hr post-load serum glucose of 140–199 mg/dL	Probiotics: 6 g/day (*Lactobacillus acidophilus, Bifidobacterium lactis, Bifidobacterium bifidum*, and *Bifidobacterium longum*, 1 × 10^9^ for each)	Placebo: maltodextrin	24 weeks	BMI	HbA_1_c, FPG↓, FIL, TG↓, TC, HDL-C, LDL-C, QUICKI, HOMA-IR, HOMA-β	*Clostridium perfringens*-to- *Escherichia coli* ratio, B*acteroides fragilis*-to-*Escherichia coli* ratio↑, *Clostridium perfringens*-to- *Bacteroides fragilis* ratio↓
					Synbiotics: comprising the mentioned probiotics with an inulin-based prebiotic			BMI	HbA_1_c↓, FPG↓, FIL↓, TG↓, TC, HDL-C, LDL-C, QUICKI↓, HOMA-IR↓, HOMA-β.	*Clostridium perfringens*-to- *Escherichia coli* ratio, B*acteroides fragilis*-to-*Escherichia coli* ratio, *Clostridium perfringens*-to- *Bacteroides fragilis* ratio
Stefanaki et al. (37)	Greece	17	No blinding	Adolescents; HbA_1_c of 5.7–6.4%, and/or FPG 100–125 mg/dL and/or 2-hr post-load serum glucose of 140–199 mg/dL	Probiotics: 450 × 10^9^ CFU for each *Streptococcus thermophilus, Bifidobacteria breve, Bifidobacteria longum, Bifidobacteria infantis, Lactobacillus acidophilus, Lactobacillus plantarum, Lactobacillus paracasei, Lactobacillus delbreuckii subspecies bulgaricus* (two times/day)	Counseling	4 months	BMI	HbA_1_c, FPG	Gut digestion: total fecal fat, cholesterol, TG, long-chain fatty acid, phospholipids, calprotectin, Eosinophil protein X, n-Butyrate concentration; Intestinal microbiome populations: *Barnesiella spp*.↓*, Faecalibacterium prausnitzii*↓, *Collinsella aerofaciens*↓, *Methanobrevibacter smithii*↓, *Escherichia coli*↑, *Anaerotruncus colihominis*↑, *Akkermansia muciniphila*↑, *Butyrivibrio crossotus*↑
Tay et al. (38)	New Zealand	26	Double	Aged 18–65 years; BMI of 30–40 kg/m^2^ or 27–40 kg/m^2^ for Indian ethnicity; HbA_1_c of 40–50 mmol/mol;	Probiotics: 6 × 10^9^ CFU *Lacticaseibacillus rhamnosus* HN001 in each capsule (in total of 90 capsules) with intermittent fasting regimen	Placebo: microcrystalline cellulose and dextrose anhydrate with intermittent fasting regimen	12 weeks	BW, BMI, waist circumference, hip circumference, WHR, neck circumference, fat (kg), fat-free mass	HbA_1_c, FPG, FIL, fasting c-peptide, TC, TG, HDL-C, LDL-C, AST, ALT, leptin, TNF-α, IL-6	N.A

* *These indicators were measured at 0, 30, 60, and 120 min*.

‡ *These indicators were measured at 0, 30, 60, 90, and 120 min*.

§ *Four articles published different outcomes of the same study and were therefore discussed together in this review*.

*No, Number; FPG, fasting plasma glucose; hr, hour; CFU, colony forming unit; SBP, systolic blood pressure; DBP, diastolic blood pressure; TC, serum total cholesterol; TG, triglycerides; HDL-C, high-density lipoprotein-cholesterol; LDL-C, low-density lipoprotein-cholesterol; N.A, Not applicable; HbA1c, glycated hemoglobin; XOS, xylooligosaccharide; BW, body weight; BMI, body mass index; HOMA-IR, homoeostasis model assessment for insulin resistance; GLP-1, Glucagon-like peptide-1; TNF-α, Tumor Necrosis Factor-α; LcS, Lactobacillus casei strain Shirota; FIL, fasting insulin level; HOMA-β, β-cell function; GOS, galacto-oligosaccharides; M-value, the mean glucose infusion rate during the final 30 min of euglycemia; SCFAs, short-chain fatty acids; TAG, triacylglyceride; FFA, free fatty acid; PYY, peptide YY; LBP, Lipopolysaccharide-binding protein; IL, Interleukin; ADA, American Diabetes Association; TMAO, trimethylamine N-oxide; TMA, trimethylamine; QUIKI, quantitative insulin sensitivity check index; WHR, waist-to-hip ratio; AST, aspartate transaminase; ALT, alanine aminotransferase; min, minutes*.

### Effects of Probiotics on Pre-diabetes

The concept of “probiotics” was first proposed by Ilya Ilyich Mechinikov, Nobel Prize in Physiology or Medicine 1908 (39). The word “probiotics” comes from the Greek word “probios,” which means “for life.” Currently, according to the Food and Agriculture Organization of the United Nations (FAO) and WHO, probiotics are defined as live microorganisms that can confer health benefits to the host when administered in adequate amounts (40).

The main advantage of probiotics is to ensure the proper balance between pathogens and bacteria that are necessary for the normal function of the organism by affecting the development of the host microbiome (41, 42). Previous molecular and genetic studies provide four mechanisms of the beneficial effect of probiotics: (1) Antagonism through the production of antimicrobial substances (43); (2) Competition with pathogens for adhesion to the epithelium and for nutrients (44); (3) Immunomodulation of the host (45); (4) Inhibition of bacterial toxin production (46). Nowadays, several studies showed that probiotics could exert antidiabetic effects, improve glucose homeostasis and delay the progression of diabetes (47–51).

The glucose metabolic outcomes of probiotics treatment for pre-diabetes are inconsistent. Naito et al. (35) found that although post-load PG levels were not significantly different between the probiotics and placebo groups, 1-h post-load PG, glycoalbumin (GA), and HbA1c levels decreased at 8 weeks compared with the baseline levels only in the probiotics group. The reduction in GA levels was statistically significantly greater in the probiotics group than in the placebo group. Interestingly, in this study, stratified analyses revealed significantly improved 1-h post-load PG and GA levels in the probiotics group compared with the placebo group among subjects with severe glucose intolerance (2-h post-load PG levels higher than the median at baseline). Another pilot study (37) conducted by Stefanaki et al. (37) showed that no difference was observed in the markers of glycemic control between the two groups after the 4-month intervention, although a minor effect was observed for fasting glucose at 1-month, probably due to the initial higher adherence to the probiotic supplements. Kassaian et al. (52) carried out a study that included probiotics, synbiotics and placebo group and published different outcomes on metabolic syndrome, lipid profiles (53), glucose and insulin metabolism (54) and gut microbiota (55). There is a significant reduction in HbA_1_c compared with the placebo group and a decrease in FPG compared with baseline. Meanwhile, homoeostasis model assessment for insulin resistance (HOMA-IR) and β-cell function (HOMA-β) were not found to be different between the probiotics group and the placebo group (35, 54). Recently, a study including 26 pre-diabetic patients demonstrated no statistically significant difference observed between the probiotic and placebo groups regarding HbA_1_c, FPG, fasting insulin or c-peptide (38).

Although previous studies have shown that probiotics can improve cholesterol levels in patients with type 2 diabetes (56), they have not been confirmed in patients with pre-diabetes (33, 35, 38). Naito et al. (35) found that after 8 weeks of intervention, the blood lipid level in the probiotics group and the placebo group increased from baseline, and did not return to baseline after elution. This increase may be related to the season change. The results showed that the serum total cholesterol (TC), high-density lipoprotein-cholesterol (HDL-C), and non-LDL-C levels in the placebo group increased significantly, while the probiotics group maintained a constant level, indicating that probiotics supplementation can suppress the rise of blood cholesterol, but there is no improvement.

Mahboobi et al. (33) found that probiotics can improve systolic blood pressure (SBP) compared with the placebo group, but there was no statistical significance after adjusting for confounding factors. However, Naito et al. (35) found no differences in blood pressure between the probiotic group and the placebo group and at any time point at baseline.

Only two studies reported group differences in intestinal microbiome populations at baseline and post-intervention. In one study by Kassaian et al. (55), the supplementation of probiotics increased B*acteroides fragilis*-to-*Escherichia coli* ratio and decreased *Clostridium perfringens*-to-*Bacteroides fragilis* ratio. In another study by Stefanaki et al. (37), the intervention group demonstrated significantly lower populations of *Barnesiella spp*. and *Butyrivibrio crossotus, Collinsella aerofaciens, Faecalibacterium prausnitzii, Escherichia coli, Akkermancia muciniphila*, compared to the control group. These populations are reported to associate with obesity (57), insulin resistance (58), gut permeability (59), and anti-inflammation (60).

### Effects of Pre-Biotics on Pre-Diabetes

Pre-biotics are non-digestible food ingredients that can be obtained from fruit, vegetables, cereals, and other edible plants. They are not metabolized or absorbed when passing through the upper gastrointestinal tract and are fermented by bacteria in the colon to enhance the growth and/or activity of beneficial bacteria (such as *Bifidobacterium* and *Lactobacillus*) (61–63). Pre-biotics can produce SCFAs, l-lactate, Carbon dioxide (CO_2_), hydrogen, methane, and other metabolites that regulate downstream metabolic process (64). As a result, pre-biotics do not promote human nutrition, but they can produce beneficial metabolism and health benefits for the host [63). A previous animal study has shown that a pre-biotic treatment decreased intestinal permeability and increased GLP-2 secretion, and reduced the hepatic expression of inflammatory and oxidative stress markers of obese and diabetic mice, as well as LPS level (65). Pre-biotics can also improve glucose levels and insulin resistance (66).

A study evaluated the effect of the pre-biotics xylooligosaccharide (XOS) in pre-diabetic subjects (34). In the gut microbiome, XOS can decrease or reverse the increase in abundance of *Howardella, Enterorhabdus*, and *Slackia*, which were observed to be higher in pre-diabetic patients. In contrast, XOS can increase the abundance of *Blautia hydrogenotrophica*, which was lower in those subjects. Although OGTT 2-h insulin response showed a tendency to decrease with XOS intervention, there were no significant differences observed in serum glucose, HOMA-IR, active GLP-1, TG, leptin, pancreatic polypeptides (PP), or the inflammatory marker TNFα. Similar to XOS, galacto-oligosaccharides (GOS) is another supplementation of pre-biotic. Canfora et al. (36) found that compared to placebo group, GOS group can affect the abundance of different microbiome populations including increased *Bifidobacterium, Prevotella oralis et rel., Prevotella melaninogenica et rel*. and decreased *Bacteroides stercoris er rel. and Sutterella wadsworthia et rel*. However, there was no significantly difference in glucose metabolism, SCFAs, gut-derived hormones, inflammation markers and insulin sensitivity.

Pre-biotics supplementation has been suggested as a strategy to reduce TMA synthetic capacity by modulating gut microbiota composition (67). However, Baugh et al. (68) found that among pre-diabetic subjects, there were no differences in fasting or post-prandial TMAO or TMA moiety concentrations after inulin intervention for 6 weeks.

### Effects of Synbiotics on Pre-diabetes

Synbiotics are a mixture of probiotics and pre-biotics. Considering the fact that probiotics are basically active in the small and large intestines, while the effects of probiotics are mainly observed in the large intestine, the combination of the two may have a synergistic effect to improve the gut health (69). The stimulation of probiotics with pre-biotics leads to the regulation of intestinal metabolic activity while maintaining the intestinal biostructure, forming beneficial bacteria, and suppressing potential pathogens in the gastrointestinal tract (70). Therefore, when combining the pre-biotic formula, it is necessary to determine the characteristics of the pre-biotics that have a beneficial effect on the probiotics (71). The use of synbiotics results in a significant increase in the levels of SCFAs, ketones, carbon disulfides and methyl acetates (72). A study (73) has reported that *Lactobacillus acidophilus* DSM20079 induced 14.5-fold more butyrate in the presence of inulin or pectin than in the presence of glucose.

Several studies revealed that synbiotics have a positive effect on blood glycemic control (74–85). Meanwhile, synbiotics have been observed to have a more significant effect on blood glycemic control and inflammation than the use of probiotics alone (86). A similar result was confirmed in the prediabetic population, Kassian et al. (52) found that synbiotic treatment improved FPG, fasting insulin levels, HbA_1_c, insulin resistance and insulin sensitivity compared with placebo, while probiotics only affected HbA_1_c. The findings suggest that a combination of probiotics and pre-biotics in the synbiotics supplementation is more effective than probiotics alone in glycemic control. Furthermore, synbiotics resulted in a higher reduction in HOMA-IR and an elevation in the QUIKI, although there was no difference in microbial abundance. However, the disadvantage of using synbiotics is that it is difficult to predict the selectivity and specificity of each component and what the resulting mechanism of action will be.

## Conclusion

Of the included studies, only three reported that the use of probiotics (37) and pre-biotics (34, 36) did not cause adverse reactions. Although many scientific reports have confirmed that gut microbiota can be beneficially modified by probiotics and/or pre-biotics to maintain glucose homeostasis, improve insulin resistance, and alleviate the development of T2D. However, in the current studies on pre-diabetes, although -biotics can alter the abundance of microbial populations, there is insufficient evidence to show significant benefits on glucose metabolism, lipid metabolism and body composition. In addition, the limited source of studies, the small sample size of each study, and the different study designs lead to inconsistent outcomes of glycemic control, pancreas islet function, changes in gut microbiota composition and other indicators between included studies. Therefore, in future researches, more and larger studies should be conducted to provide favorable evidence for -biotics to improve pre-diabetes, thereby providing a new therapeutic tool to prevent and delay the development of pre-diabetes to T2D.

## Data Availability Statement

The original contributions generated for the study are included in the article/supplementary material, further inquiries can be directed to the corresponding author.

## Author Contributions

XW and JY conceived the idea and drafted the initial manuscript. XW, JY, and XQ designed the review. QW, ML, and DZ reviewed scoping searches and contributed to the methodological development of the review. All the authors (XQ, QW, ML, and DZ) revised the manuscript. All the authors have given approval of publishing. QC is the review guarantor.

## Conflict of Interest

The authors declare that the research was conducted in the absence of any commercial or financial relationships that could be construed as a potential conflict of interest.

## Abbreviations

T2D: type 2 diabetes
IFG: impaired fasting glucose
IGT: impaired glucose tolerance
WHO: the World Health Organization
ADA: the American Diabetes Association
HbA_1_c: glycated hemoglobin
IDF: the International Diabetes Federation
SCFAs: short-chain fatty acids
GLP: glucagon-like peptide
LPS: Lipopolysaccharides
TLR: toll-like receptor
eCB1: endocannabinoid receptors
BA: bile acids
FXR: farnesoid X receptor
TGR5: Takeda G protein-coupled receptor 5
FGF19: fibroblast growth factor-19
TMA: trimethylamine
TMAO: trimethylamine N-oxide
FPG: fasting plasma glucose
RCTs: randomized controlled studies
PRISMA-P: the preferred reporting items for systematic review and meta-analysis protocols
FAO: the Food and Agriculture Organization of the United Nations
GA: glycoalbumin
HOMA-IR: homoeostasis model assessment for insulin resistance
HOMA-β: β-cell function
TC: total cholesterol
HDL-C: high-density lipoprotein-cholesterol
SBP: systolic blood pressure
CO_2_: Carbon dioxide
XOS: xylooligosaccharide
PP: pancreatic polypeptides
GOS: galacto-oligosaccharides
QUIKI: quantitative insulin sensitivity check index.
